# South East Asian Nutrition Surveys (SEANUTS) II – a multi-country evaluation of nutrition and lifestyle indicators in children aged 12 years and below: rationale and design

**DOI:** 10.1017/S1368980024000910

**Published:** 2024-04-19

**Authors:** Serene Yaling Tan, Bee Koon Poh, Rini Sekartini, Nipa Rojroongwasinkul, Thuy Nga Tran, Jyh Eiin Wong, Dian Novita Chandra, Tippawan Pongcharoen, Khanh Van Tran, Lucas Actis-Goretta, Marlotte M Vonk, Swee Ai Ng, Panam Parikh, Ilse Khouw, SEANUTS II Study Group

**Affiliations:** 1 FrieslandCampina, Amersfoort, The Netherlands; 2 Centre for Community Health Studies (ReaCH), Faculty of Health Sciences, Universiti Kebangsaan Malaysia, Kuala Lumpur, Malaysia; 3 Cipto Mangunkusumo General Hospital, Universitas Indonesia, Depok, Indonesia; 4 Food and Nutrition Academic and Research Cluster, Institute of Nutrition, Mahidol University, Nakhon Pathom, Thailand; 5 Department of Micronutrients, National Institute of Nutrition, Hanoi, Vietnam

**Keywords:** Children, Health and lifestyle, Malnutrition, Nutrition survey, Southeast Asia

## Abstract

**Objective:**

To assess the nutritional status, growth parameters and lifestyle behaviours of children between 0·5 and 12 years in nationally representative samples in Malaysia, Indonesia, Thailand and Vietnam.

**Design:**

A cross-sectional study was conducted in the four countries, between May 2019 and April 2021. Data collected can be categorised into four categories: (1) Growth – anthropometry, body composition, development disorder, (2) nutrient intake and dietary habits – 24-h dietary recall, child food habits, breast-feeding and complementary feeding, (3) socio-economic status – food insecurity and child health status/environmental and (4) lifestyle behaviours – physical activity patterns, fitness, sunlight exposure, sleep patterns, body image and behavioural problems. Blood samples were also collected for biochemical and metabolomic analyses. With the pandemic emerging during the study, a COVID-19 questionnaire was developed and implemented.

**Setting:**

Both rural and urban areas in Malaysia, Indonesia, Thailand and Vietnam.

**Participants:**

Children who were well, with no physical disability or serious infections/injuries and between the age of 0·5 and 12 years old, were recruited.

**Results:**

The South East Asian Nutrition Surveys II recruited 13 933 children. Depending on the country, data collection from children was conducted in schools and commune health centres, or temples, or sub-district administrative organisations.

**Conclusions:**

The results will provide up-to-date insights into nutritional status and lifestyle behaviours of children in the four countries. Subsequently, these data will facilitate exploration of potential gaps in dietary intake among Southeast Asian children and enable local authorities to plan future nutrition and lifestyle intervention strategies.

Over the past decades, the world has shown strong socio-economic development, which has had both positive and negative effects on almost every aspect of human life, including technological advancements and transformation in lifestyle habits^(1)^. In Asia, rapid industrialisation has brought about significant lifestyle and dietary changes^(1)^, and many countries are experiencing nutrition transition towards a diet with increased intake of meats, foods higher in fats and sugars and reduced physical activity^(2)^. This, in turn, brings direct effects on children’s nutrition and health status^(2)^. Poor nutrition in children is a major risk factor for conditions such as stunting and wasting, overweight and obesity, cognitive impairment, poor school performance, long-term chronic illness, subsequent reduced adult income and consequently has a negative impact on national Gross Domestic Product and productivity^(3)^.

The 2020 Global Nutrition Report shows that, despite the significant steps the world has taken towards improving nutrition and associated health burdens over recent decades, poor nutrition remains a large-scale and universal problem with 87 % of countries still facing a serious burden of at least two forms of malnutrition (stunting/overweight and anaemia or stunting and overweight), while 26 % faced all three forms of malnutrition^(4)^. Children are one of the most affected groups. In 2019, 144 million children experienced stunting, 47 million children wasting and 38 million children were overweight globally^(5)^. One of the sustainable development goals set by UN is to reduce the number of children with stunting to 82·5 million by 2030 (50 % reduction from 165 million in 2012). According to UN, more intensive efforts are required to achieve this goal, as there was only a 2 % reduction between 2015 and 2019^(5)^. Double burden of malnutrition is experienced in many developing countries, particularly in the Asia Pacific region, where undernutrition coexist along with overweight/obesity and other nutrition-related non-communicable diseases^(6)^. A varying prevalence of double burden of malnutrition has been observed in Southeast Asian families, with double burden of malnutrition ranging from 5·0 % in Vietnam to as high as 30·6 % in Indonesia^(6)^.

Large-scale surveys that comprehensively address the present nutrition situation among Southeast Asian children are scarce^(7)^. Moreover, previous national surveys in the region included only data on growth, infant and young child feeding practices and dietary intakes^(8,9)^. These surveys either focused on children <5 years or between 6 and 17 years old. Hence, there are insufficient up-to-date nutritional data especially for children aged between 0·5 and 12 years old living in Southeast Asia. Therefore, there is an urgent need for updated quantitative data that would identify the nutrition gaps in a broader age group.

In addition to analysing the children’s nutritional status and dietary intake, lifestyle behaviours such as physical activity are also vital for children’s growth, motor and cognitive development and also reduce risk of chronic diseases^(10)^. Children who are engaged in higher physical activity have been shown to have a more positive psychosocial health and improved motor development^(11)^. However, it has been reported in many Western and Asian countries including Singapore, China and South Korea that children are not meeting the healthy movement recommendations^(11)^. Besides being physically active, sleep is also another component that is equally important for a child’s development as well as physical and mental health^(12)^. In general, school-aged children are sleeping less now compared with decades ago, largely due to more electronic media screen time^(12)^. It is critical especially for children to have adequate sleep as long-term sleep loss could cause problems relating to the child’s health, poor attention span and less successful academic performance^(13)^.

The recent coronavirus disease 2019 (COVID-19) outbreak also brought about lifestyle shifts in school-aged children such as increase in virtual education, loss of social interactions which challenges their well-being and development^(14)^. It was reported that school closures could have an impact on the nutrition, education and mental health of children, especially those in monetary-poor households^(15)^.

The South East Asian Nutrition Surveys (SEANUTS) I, conducted in 2010/2011, was one of the largest multi-center nutrition and health studies conducted in Southeast Asia, where data of nearly 17 000 children from Malaysia, Indonesia, Thailand and Vietnam were collected^(16)^. The four countries were chosen due to the high prevalence of malnutrition^(6)^. As a follow-up to SEANUTS I, the primary aim of SEANUTS II was to obtain updated data on the nutritional and health status of children in the same four countries. In addition, with the on-going COVID-19 pandemic, a questionnaire was developed to study the impact of COVID-19 on the child’s dietary intake as well as parents’ and or main caregiver’s work status and household expenditure patterns.

To our knowledge, SEANUTS II is the first study that will generate insights focused on dairy intake, metabolomic profile as well as physical fitness levels of children in the participating countries. The main objective was to assess the nutritional status, growth parameters and lifestyle behaviours of children between 0·5 and 12 years in a nationally-representative population in each of the four countries. This paper describes the general study design and methodology of SEANUTS II and discusses some of the challenges faced during the course of the study.

## Methods and materials

### Study design

SEANUTS II is a cross-sectional study conducted in four countries: Malaysia, Indonesia, Thailand and Vietnam in both urban and rural areas. Study protocols were prepared by all principal investigators involved in close collaboration with FrieslandCampina (Table 1). The methodology was standardised wherever feasible to allow for comparison of results among countries.

**Table 1:** Principal investigators participating in the South East Asian Nutrition Surveys II

Country	Institute	Principal investigator
Malaysia	Universiti Kebangsaan Malaysia	Prof Dr Poh Bee Koon
Indonesia	Universitas Indonesia	Prof Dr Rini Sekartini
Thailand	Mahidol University	Assoc Prof Dr Nipa Rojroongwasinkul
Vietnam	National Institute of Nutrition	Assoc Prof Dr Tran Thuy Nga

### Study participants

In order to participate in the study, apparently healthy participants had to be within the age of 0·5–12 years and citizen of the studied country. Exclusion criteria for participation were signs of physical disability, genetic, cardiovascular or respiratory illness that limited physical activity. Also, in case a subject was unwell on the day of measurement, they were excluded. Only one of the siblings was recruited to participate in the study. In total, the study recruited 13 933 children.

### Sample size calculation

The total sample size of each country was based on the potential occurrence of stunting and/or overweight, and/or anaemia, and/or vitamin A deficiency, and/or vitamin D deficiency, and/or zinc deficiency, depending on national nutritional issues. Therefore, the estimated sample size for each country was based on the national prevalence data of the above-mentioned issue(s) (Table 2).

**Table 2:** National prevalence data on obesity, stunting, anaemia, vitamin A, D and zinc deficiency

	Obesity	Stunting	Anaemia	Vitamin A deficiency	Vitamin D deficiency	Zinc deficiency
Malaysia	11·8 %^(17)^	–	–	–	–	–
Indonesia	3·7–8·0 %^(18)^	30·7–37·2 %^(18)^	38·2–48·1 %^(19)^	–	–	–
Thailand	6·9–8·0 %^(20)^	4·1–8·4 %^(20)^	9·0–18·4 %^(20)^	–	27·7–45·6 %^(20)^	–
Vietnam	–	15·6–24·3 %^(21,22)^	11·3–23·4 %^(21)^	5·0–14·2 %^(8,21)^	48·0–53·0 %^(21,23)^	69·4–81·5 %^(22,24)^

The formula used for calculating the sample size was:

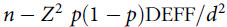




where ‘*n*’ is the total number of participants, ‘*Z*’ is the confidence level (*α* = 0·05 and *Z* = 1·96), ‘*p*’ is the prevalence (%) of nutritional status of interest (see Table 2), ‘DEFF’ refers to the estimated design effect, estimated at 2 and ‘*d*’ is the tolerable error. Finally, possible non-response rate per country was considered. This resulted in a sample size of 3864 subjects for Malaysia, 7595 subjects for Indonesia, 3540 for Thailand and 4088 for Vietnam.

The total number of participants recruited in each county by age and the total completeness rate per country is presented in Table 3.

**Table 3:** Overview of the number of participants recruited in each country by age

Age (years)	Malaysia	Indonesia	Thailand	Vietnam
0·5–0·9	78	425	297	350
1·0–3·9	558	1411	1212	1278
4·0–6·9	926	537	881	789
7·0–12·9	1427	1092	1088	1584
Total	2989	3465	3478	4001
Completeness rate	77 %	46 %	98 %	98 %

#### Sampling method and recruitment

A multi-stage clustered sampling approach was employed for all four countries. In Malaysia, it was based on the 2010 national population census stratified by region and age^(25)^. Six regions (Northern, Southern, East Coast and Central regions of Peninsular Malaysia as well as Sabah and Sarawak) were identified, and one urban and one rural district were selected per region. In the second stage, enumeration blocks were randomly selected from where subjects were recruited.

In Indonesia, sample selection was conducted in forty-six regencies across thrity-four provinces, selected based on 2010 national population data by the Indonesian National Bureaus of Statistics^(26)^. Proportional sampling was applied for regencies selection. From each selected regency (stage one), one district was randomly selected (stage two), followed by the selection of one sub-district (stage three) and one hamlet (stage four), In stage five, children under 7 years were randomly selected from a list of households provided by the head of the hamlet, whereas the 7·0–12·9-year-old children were randomly identified from the elementary school list provided by the local sub-district government. For families with multiple eligible siblings, only the youngest was selected.

In Thailand, the samples were selected randomly in four regions (Central, North Eastern, Northern and Southern) and Bangkok using probability proportional to size procedure based on 2017 national population data by the Department of Provincial Administration, Ministry of Interior^(27)^. Then, provinces within each region were sampled, second, a district per province was selected and then urban and rural enumeration areas were selected. Within each enumeration areas, a random sample of households was drawn to recruit the subjects.

Lastly, in Vietnam, sampling was done in four regions (Northern Mountainous and Central Highlands; Red River Delta; North Central and Central Coastal; Southeast and Mekong River Delta) using a multi-stage cluster systematic random sampling method based on the 2019 national population data by the General Statistics Office, Ministry of Planning and Investment of Vietnam^(28)^. In the first stage, one city and two rural provinces were selected in each region and considered as primary sampling units. The study population was recruited from four big cities and eight rural provinces (twelve cities/provinces in total). In the second stage, in each primary sampling unit, three community-based clusters and three school-based clusters were selected by probability proportional to size and considered as secondary sampling units. In the third stage, children were randomly selected from the children list in each community-based cluster (total of thirty-six communes), and children in primary schools were randomly selected from the children list in each school (total of thirty-six schools).

In all countries, recruitment included both urban and rural areas, based on the respective local definitions by Department of Statistics or Ministry of Interior: in Malaysia, urban areas are areas with a population of ≥ 10 000; Indonesian urban areas have a population of ≥ 8500; Thailand defines urban areas as municipalities with a population of ≥ 7,000 and in Vietnam, urban areas have a population of ≥ 5,000.

### Ethics and informed consent

The study protocol, questionnaires, informed consent forms, recruitment materials and any written information, including all amendments provided to the participants and/or parents or main caregivers prior to participation in the study, were reviewed and approved by the Research Ethics Committee of the participating institutes, which is presented in Table 4.

### Data collection and management

Prior to the initiation of the data collection, field staff from all countries were trained by the same trainer for all assessment methods and study procedures including anthropometric measurements, clinical examinations, interview techniques, field management and data management. Each of the local teams consisted of staff members with nutrition and/or biomedical science/physical activity knowledge. Standard operating procedures and work instructions were designed, and training sessions were conducted to minimise intra- and inter-observer variations.

The countries used local mobile field team(s) to visit the identified regions for data collection. Data were collected between May 2019 and April 2021 and was done using either paper questionnaires or direct input into the electronic data management system, specifically designed to capture all data except for the 24-h dietary recall, biochemistry, accelerometer and body composition data. Data collection in Malaysia and Indonesia was done through home visits for children aged <6 years and in schools for participants aged 7–12 years. In Thailand, data collection was conducted at schools, temples and sub-district administrative organisations for children between 0·5 and 12 years, while in Vietnam it was conducted at schools and commune health centres, also for children aged 0·5–11 years. Data accuracy was assured by double data entry for at least 10 % of data and by performing source data verification for at least 20 % of data collected via paper questionnaires. The sponsor conducted monitoring visits to all four countries.

### Assessments

The following assessments were conducted either in all children or in a sub-sample. An overview of the study parameters and methodology for each country is presented in Table 5.

**Table 4:** Overview of ethics committee and number for each country

	Malaysia	Indonesia	Thailand	Vietnam
Ethics committee	Research Ethics Committee of Universiti Kebangsaan Malaysia	Research Ethics Committee of RSCM Hospital and Faculty of Medicine, Universitas Indonesia	Mahidol University Central Institutional Review Board (MU Central-IRB)	Research Ethics Committee of the National Institute of Nutrition
Ethics number	JEP-2018–569	0031/UN2.F1/ETIK/2019 Protocol nr: 19-01-0046	COA nr MU-CIRB 2019/143·0209	765/VDD-QLKH in 2019 Protocol nr: 2720/ QD-VDD in 2019

**Table 5:** Overview of South East Asian Nutrition Surveys (SEANUTS) measurements, materials and methods for Malaysia, Indonesia, Thailand and Vietnam

	Malaysia	Indonesia	Thailand	Vietnam
Study design	Cross-sectional	Cross-sectional	Cross-sectional	Cross-sectional
Data collection	May 2019 – March 2020	September 2019 – March 2020	January – December 2020	September 2020 – April 2021
Age of subjects	6 months – 12·9 years	6 months – 12·9 years	6 months – 12·9 years	6 months – 11·9 years
Sampling method	Population-based, multistage cluster sampling; Home (0·5–6·9 years) and School based (7–12·9 years)	Population-based, multistage cluster sampling; Home (0·5–6·9 years) and School based (7–12·9 years)	Population-based, multistage cluster sampling; Household (0·5–12·9 years)	Population-based, multistage cluster sampling; Commune Health Center (0·5-<6 years) and School based (6–11·9 years)
Data entry	Paper → transfer to VIEDOC	Paper → transfer to VIEDOC	– Questionnaires → enter directly to VIEDOC – Measurement record form (paper) → transfer to VIEDOC	– Questionnaires → enter directly to VIEDOC – Measurement record form (paper) → transfer to VIEDOC
Weight	SECA 354 (Infant); SECA 874 (Child)	SECA 334 (Infant); SECA 874 (Child)	SECA 834 (Infant); SECA 874 (Child)	SECA 334 (Infant); SECA 874 (Child)
Height (standing)	SECA 210 and 417 (Infantometer); SECA 213 (Stadiometer)	SECA 417 (Infantometer); SECA 217 (Stadiometer)	SECA 417 (Infantometer); SECA 217 (Stadiometer)	SECA 417 (Infantometer); SECA 217 (Stadiometer)
Height (sitting)	SECA 213 and sitting box	SECA 217 and sitting box	Nil	SECA 217 and sitting box
Circumferences (mid-upper arm, head, waist and hip)	Lufkin tape W606PM Head: SECA 212	Lufkin tape W606PM	Mid-upper arm, waist: Rosscraft tape Head: SECA 201	Waist and hip: SECA 201 Mid-upper arm and head: UNICEF tape
Skinfolds (triceps and subscapular)	Harpenden	Harpenden	Holtain	Harpenden
Breadths (elbow, wrist, knee)	Holtain Bicondylar caliper	Holtain Bicondylar caliper	Nil	Holtain Bicondylar caliper
Body composition	InBody 120 (3–12·9 years old)	InBody 120 (7–12·9 years old)	InBody 120 (6–12·9 years old and sub-group of 4–5·9 years old selected for blood sample)	InBody 120 (6–11·9 years old)
Blood pressure	Welch Allyn ProBp 3400	Tensimeter digital Omron Hem1300	Nil	Rossmax AW150
Dietary assessment	−1-day 24-hr recall (All) – BCF (0·5–1·9 years old) – CFH (2–12·9 years old) – Dairy Consumption Questionnaire (2–12·9 years old) – FIQ (All)	−1-day 24-hr recall (All) – BCF (0·5–1·9 years old) – CFH (2–12·9 years old) – Dairy Consumption Questionnaire (2–12·9 years old) – FIQ (All)	−1-day 24-hr recall (All) – BCF (0·5–1·9 years old) – CFH (2–12·9 years old) – Dairy Consumption Questionnaire (2–12·9 years old);	−1-day 24-hr recall (All) – BCF (0·5–1·9 years old) – CFH (2–11·9 years old) – Dairy Consumption Questionnaire (2–11·9 years old) – FIQ (All)
Physical activity	– Accelerometer (GENEActiv) (Sub-group: 3–12·9 years old) – Fitness test (Modified 15-meter Shuttle Run, Standing Long Jump, Handgrip Strength (JAMAR Plus hand dynamometer), Sit-up Test, V-Sit and Reach) (Sub-group: 6–12·9 years old) – PAQ (All)	– Accelerometer (GENEActiv) (Sub-group: 7–12·9 years old) – Fitness test (Modified 15-meter Shuttle Run, Standing Long Jump, Handgrip Strength (JAMAR Plus hand dynamometer), Sit-up Test, V-Sit and Reach) – PAQ (7–12·9 years old)	– Accelerometer (GENEActiv) (Sub-group: 6–12·9 years old selected for blood sample) – Fitness test (Modified 15-meter Shuttle Run, Standing Long Jump, Handgrip Strength (JAMAR Plus hand dynamometer), Sit-up Test, V-Sit and Reach) (Sub-group: 6–12·9 years old) – PAQ (All)	– Accelerometer (GENEActiv) (Sub-group: 4–11·9 years old selected for blood sample) – Fitness test (Modified 15-meter Shuttle Run, Standing Long Jump, Handgrip Strength (JAMAR Plus hand dynamometer), Sit-up Test, V-Sit and Reach) (Sub-group: 6–11·9 years old) – PAQ (All)
Child development	– Ages and Stages Questionnaire, gross motor domain (<6 years old)	– DCDQ (7–12 years old) – M-CHAT (18–36 months) – Abbreviated Conners Rating Scale (36–72 months)	Nil	Nil
Sleep pattern	– BISQ (<3 years old) – CSHQ (>3 years old) – PSQI (10–12 years old)	– BISQ (<3 years old) – SDSC (>3 years old)	Nil	– SAQ (All)
Socio-economic	SES (All)	SES (All)	SES (All)	SES (All)
Health status of child	CHE (All)	CHE (All)	CHE (All)	CHE (All)
Perception of ideal body size	BIQ (10–12·9 years old)	BIQ (7–12·9 years old)	BIQ (10–12·9 years old)	BIQ (10–11·9 years old)
Ultraviolet exposure	UVB Badge: Polysulphone film badge (Sub-group: 4–12·9 years old selected for blood sample)	UVB Badge: Polysulphone film badge (Sub-group: 7–12·9 years old)	UVB Badge: Polysulphone film badge (Sub-group: 6–12·9 years old selected for blood sample)	UVB Badge: Polysulphone film badge (Sub-group: 4–11·9 years old selected for blood sample)
Blood	– Finger prick (Sub-group: <4 years old), only Hb – Venous blood (Sub-group: 4–12·9 years old)	– Venous blood (Sub-group: all age groups)	– Heel/Finger prick (Sub-group:1/3 of <4 years old), only Hb – Venous blood (Sub-group: 1/3 of 4–12·9 years old)	– Finger prick (Sub-group: <4 years old), only Hb – Venous blood (Sub-group: 4–11·9 years old)
Hb	HemoCue Hb 201+ (Sub-group: <4 years old)	SLS-Hemoglobin	HemoCue Hb 201+ (Sub-group: 1/3 of <4 years old)	HemoCue Hb 301 (Sub-group: <4 years old)
Full blood count	Flow Cytometry	Flow Cytometry	Fluorescent Flow Cytometry, including Hb (Sub-group: 1/3 of 4–12·9 years old)	Fluorescent Flow Cytometry (Sub-group: 4–11·9 years old)
Iron	Spectrophotometry	Ferene	Nil	Nil
Ferritin	Spectrophotometry	CMIA	CMIA	ELISA
Transferrin receptor	Nil	Nil	ELISA	Nil
Transferrin saturation	Spectrophotometry	Nil	Nil	Nil
Alpha-1-acid glycoprotein	ELISA	Nephelometry	ELISA	ELISA
C-reactive protein	Spectrophotometry	Immunoturbidimetric-CMIA	Immunoturbidimetric Assay	Immunoturbidimetric Assay
Vitamin B_12_	ECLIA	CMIA	CMIA	LC-MS/MS
Vitamin A	Liquid-liquid Extraction	HPLC	HPLC	HPLC
Vitamin D (serum 25- hydroxyvitamin D)	ECLIA	CLIA	LC-MS/MS	LC-MS/MS
Zinc	Nil	ICP-MS	ICP-MS	AAS
Fasting blood glucose	Spectrophotometry	Hexokinase-CMIA	Nil	Spectrophotometry
Lipid profile	Spectrophotometry	CMIA	Nil	Spectrophotometry
Parathyroid hormone (PTH)	Nil	Nil	CMIA	CMIA
Hb typing	Nil	Nil	Capillary Electrophoresis	Nil
Metabolomics	Biocrates Absolute*IDQ* p180 Kit	Biocrates Absolute*IDQ* p180 Kit	Biocrates Absolute*IDQ* p180 Kit	Biocrates Absolute*IDQ* p180 Kit

AAS, atomic absorption spectroscopy; BCF, Breastfeeding and Complementary Feeding Questionnaire; BIQ, Body Image Questionnaire; BISQ, Brief Infant Sleep Questionnaire; CFH, Child Food Habits Questionnaire; CHE, Child Health Status/Environmental Factor Questionnaire; CLIA, chemiluminescence immunoassay; CMIA, chemiluminescent microparticle immunoassay; CSHQ, Child Sleep Habit Questionnaire; DCDQ, Development Disorder Questionnaire; ECLIA, electrochemiluminescence immunoassay; FIQ, Food Insecurity Questionnaire; ICP-MS, inductively coupled plasma-MS; immuno-CL, immuno-chemiluminescence; LC-MS/MS, liquid chromatography triple quadrupole MS; M-CHAT, modified checklist for autism in toddlers; PAQ, Physical Activity Questionnaire; PSQI, Pittsburgh Sleep Quality Index; SAQ, Sleep Assessment Questionnaire; SDSC, Sleeping Disturbance Scale for Children; SES, Socio-economic Questionnaire; SLS, sodium lauryl sulphate.

### Anthropometric and body composition measurements

Body weight was measured using SECA digital weighing scale to the nearest 0·02 kg for children aged less than 2 years and 0·05 kg for older children. Recumbent length was measured for children aged <2 years using SECA infantometer while standing height was measured for children aged ≥2 years using a SECA stadiometer, both measured to the nearest 0·1 cm. For weight measurements, children were weighed in minimal clothing without shoes. For height measurements, children were measured standing straight and bare footed with the child’s head in the Frankfort plane. The appropriate WHO age- and sex-specific *z*-scores were calculated using the measured weight and height^(29)^.

BMI was calculated as weight (kg)/height squared (m^2^). Body omposition was measured in school-aged children using Bioelectrical Impedance Analysis, InBody 120 (InBody Co., Ltd).

Mid-upper arm circumference, head circumference, waist circumference and hip circumference were measured using the Lufkin/Rosscraft/UNICEF tape and SECA 201/212 to the nearest 0·1 cm.

Triceps and subscapular skinfolds were measured with skinfold caliper (Harpenden/Holtain) to the nearest 0·1 mm using a standardised anthropometry procedure^(30)^. The skinfold readings were compared with the WHO growth standards^(29)^.

Elbow, wrist and knee breadths were measured with small sliding caliper (Holtain) to the nearest 0·1 cm. Breadths were measured only in Indonesia and Malaysia.

All anthropometric measurements were carried out in duplicates. A third measurement was done if difference between the two measurements was 0·1 kg or more for weight, 0·5 cm or more for height/length, mid-upper arm circumference, waist circumference and head circumference or 2 mm or more for skinfold measurements. The mean of two measurements or median of three measurements was used as the final value.

### Socio-economic and general health status

The socio-demographic information of the child and the parents or main caregivers was collected using the Socio-economic Questionnaire. Child’s information included date of birth, birth weight, ethnicity and sex. Parents’ information included age, height and weight, ethnicity, education level, occupation, and total household monthly income.

The Child Health Status/Environmental Factor Questionnaire comprised of two parts: (A) information on child health, including general health problems, medication, hearing or speech problems, chronic diarrhoea, constipation, dental problems and presence of milk allergy or intolerance and (B) information on environmental factors that are related to child health, including safety of drinking water source, toilet facilities, rubbish disposal and hand-washing habits^(31,32)^.

The Socio-economic Questionnaire and Child Health Status/Environmental Factor Questionnaire were self-administered by parents or main caregivers in Malaysia and done via face-to-face interview in the other countries.

### Dietary intake and food habits

Dietary intake was assessed using a one-day 24-h recall and conducted on the day of data collection. The 24-h recall was collected via: (1) parent-proxy report by mother or main caregiver through face-to-face interview for children aged 0·5–10 years and (2) combination of child self-report and parent-proxy through face-to-face interview for children aged 10–12 years. In order to improve the accuracy of reporting, household measures and photographs of food portion sizes were prepared to assist recall. In Thailand, parents or main caregivers were also asked to record all foods consumed the day before data collection for better memory support.

The 24-h dietary recall was then converted into nutrient intakes per day as well as per meal using nutrition analysis software with local food composition database. Regarding breastmilk consumption, estimating the volume of breastmilk intake for those below 12 months of age was based on a fixed volume set as total daily intake, whereas for those older than 12 months, it was a fixed volume per feed^(33–35)^. Data from the 24-h dietary recall was further supplemented with following questionnaires: Child Food Habit^(16,36)^, Breast-feeding and Complementary Feeding^(37)^ and Food Insecurity Questionnaire^(38,39)^.

The Child Food Habit questionnaire obtained descriptive data of children aged 2–12 years on meal patterns (breakfast, lunch and dinner), snacking patterns, unhealthy eating habits, fruit and vegetables consumption, whole grains food consumption and milk and dairy consumption. Breast-feeding and complementary feeding patterns among children between 0·5 and 2 years old were determined using the Breast-feeding and Complementary Feeding questionnaire. The Food Insecurity Questionnaire was used to assess four levels of food insecurity with increasing severity – food secure, household food insecure, individual or adult food insecure and child hunger. This questionnaire is derived from Radimer/Cornell Hunger and Food Insecurity instrument^(38)^ that has been validated^(39)^. Thailand did not implement this Food Insecurity Questionnaire.

### Physical activity, fitness and ultraviolet B exposure

Physical activity and sleep behaviour were assessed using both subjective and objective methods. A Physical Activity Questionnaire^(40,41)^ was administered to assess physical activity of the children, sedentary behaviour, screen time, active transport, parent and peer support and home and community environment. The Physical Activity Questionnaire was administered only in school-aged children in Indonesia, whereas all the other countries used Physical Activity Questionnaires specific for the age of the participants.

A Sleep Assessment Questionnaire was used to assess sleep duration, sleep pattern and sleep quality of children in Vietnam, with two questions added to assess for anxiety and depression^(42,43)^. In Malaysia and Indonesia, the Brief Infant Sleep Questionnaire was used. In addition, Malaysia also used the Child Sleep Habit Questionnaire and the Pittsburgh Sleep Quality Index for children who were able to self-report. The Sleep Disturbance Scale for Children was also used in Indonesia. Although Thailand did not implement any sleep questionnaire, two questions related to sleep duration were added in the Physical Activity Questionnaire for children aged 7 years and above.

In a sub-sample of participants (3–12 years for Malaysia, 6–12 years for Indonesia and Thailand and 4–11 years for Vietnam), a tri-axial, waterproof accelerometer GENEActiv (ActivInsights Ltd) was used to assess physical activity and sleep patterns. The children wore the accelerometer on their non-dominant wrist for seven consecutive days. The accelerometer measures movement is then used to estimate intensity of physical activity, sedentariness and sleep.

Measurements of physical fitness including cardiorespiratory endurance, muscular strength, muscular endurance and flexibility were conducted amongst school-aged children. The fitness tests included: (1) modified 15-m shuttle run (assess cardiorespiratory endurance),^(44)^ where children ran continuously between two lines 15 m apart in time with an audio signal after which pulse rate was recorded; (2) standing long jump (assess lower body explosive strength)^(45)^, where children had to stand behind a marked line with feet slightly apart and jump forward with swinging of the arms and bending of knees motion; (3) handgrip strength (assess upper body isometric strength)^(46)^, where children were seated with a straight posture, with feet flat on the ground and elbow flexed at 90^o^. The children were then instructed to squeeze the JAMAR Plus hand dynamometer (JLW Instruments) with as much force as possible; (4) sit-up (assess abdominal strength and endurance), where children were asked to lie in a supine position with knees bent at 90^o^, with feet flat on the floor and legs slightly apart. The children were then instructed to lift the upper body until the hands touched the thighs, then curl back down, continuing this action for 30 s and the total number of sit-ups performed was recorded and lastly (5) V-sit and reach (assess flexibility of the lower back and hamstring muscles)^(47)^, where children sat on the floor with the measuring line between their legs with the soles of their feet placed behind the baseline, heels 20–30 cm apart. Children were then instructed to slowly reach forward as far as possible, keeping the fingers on baseline and feet flexed.

In a sub-sample of children (10–30 %) who had their blood withdrawn, exposure to sunlight was assessed through UVB exposure using polysulphone dosimeter film badges. Each child was given one UVB badge, which was worn on their outdoor clothing for four days. The parents or main caregivers were instructed to keep the UVB badge in a thick envelop provided to keep it from being exposed to sunlight before and after use in order to maintain its stability.

### Body image and child development

The Body Image Questionnaire consisted of figure rating scale with two questions to assess girls and boys perception of their actual body size and ideal body size^(48)^.

In Indonesia, the Development Disorder Questionnaire, Modified Checklist for Autism in Toddler questionnaire and Conner’s Rating Scale were administered to assess for any coordination disorders, risk of autistic spectrum disorder and attention deficit hyperactivity disorder respectively. In Malaysia, gross motor development was assessed in children below 6 years using the Ages and Stages Questionnaire^(49)^.

### Biochemical analysis, metabolomics and blood pressure

Venous blood of approximately 7–15 ml was collected from a random sub-sample of children aged ≥4 years in Malaysia, Thailand and Vietnam. In Indonesia, blood sample was collected from all age groups. The blood samples were collected after a 12-h overnight fast by a trained phlebotomist. The collected blood samples were kept in a standard storage box with an ice pack and transported immediately to an accredited laboratory for analysis according to their standard methods. Among sub-sample of children aged 0·5–4 years in Malaysia, Thailand and Vietnam, 10–20 μl of blood was collected via fingerpick to measure Hb levels. Table 5 provides an overview of biochemical analyses for each country.

In approximately 400 children, metabolomic analysis will be conducted using the Biocrates Absolute*IDQ* p180 kit using the liquid chromatography-mass spectroscopy// mass spectroscopy methodology. Over 180 metabolites in the classes of amino acids, biogenic amines, hexose, acylcarnitines and sphingolipids will be quantified.

Systolic and diastolic blood pressure was measured in school-aged children with an automated blood pressure monitor with appropriate cuff size, except in Thailand.

### COVID-19 questionnaire

To understand the impact of the pandemic on economic situation, lifestyle and food habits, an additional questionnaire was developed and implemented in all countries. Malaysia was the first to implement the questionnaire in June 2020, followed by Thailand in July 2020, and both Indonesia and Vietnam were the last to implement in September 2020. The COVID-19 questionnaire assessed parents’ and/or main caregiver’s work status, family income and household food expenditure pattern during the local COVID-19 lockdown. The questionnaire also focused on the child’s dietary intake, such as any changes in type of food, portion size and snacks taken. It also evaluated the amount of physical activity and screen time of the child. Malaysia and Indonesia implemented the COVID-19 questionnaire after data collection was completed to a sub-group of children (∼25 % and ∼43 % of recruited participants in Malaysia and Indonesia, respectively), Thailand implemented to ∼80 % of the children while all children in Vietnam completed this questionnaire. In addition to the COVID-19 questionnaire, the same sub-group of children in Malaysia and Indonesia also repeated the Child Food Habit questionnaire, Food Insecurity Questionnaire and Physical Activity Questionnaire.

### Statistical analysis

Statistical analysis was performed using IBM SPSS Statistics version 23·0 for Windows (IBM Corp., Armonk NY, USA) with complex samples module. Descriptive analysis was performed and presented as mean and se. ANCOVA after adjusting for covariates was used to examine the factors influencing growth and nutritional status of children. Generalised linear model was used to build the regression models of the study, to examine the interrelationship between parameters, taking confounding factors into account. Bivariate analysis was carried out between the parameters of interest using *χ*^2^ test for categorical outcomes or correlation test for numerical outcomes. Throughout the study, a *P* value <0·05 was considered to be statistically significant when applying two-sided testing. A comprehensive statistical analysis plan was developed and endorsed by all principal investigators before finalising the database.

## Discussion

Although SEANUTS II was designed and implemented before the current coronavirus pandemic, the need for identifying the scope and scale of nutritional issues has never been more urgent than in this time of unparalleled uncertainty. With the expected rise in malnutrition, there is a heightened need for screening communities, food and nutrition surveillance and identify the potentially vulnerable populations to improve targeting and program designing. A multi-sectorial approach to support and protect nutritionally vulnerable groups is now more critical than ever. SEANUTS II entails a unique opportunity to provide this essential information to governments and relevant stakeholders, aiming to enforce public health policies and to provide new insights for nutritional research. A strong asset of SEANUTS II is the four-country set-up where almost identical protocols were implemented.

SEANUTS II has included more measurements and collected more in-depth information, compared with its predecessor (SEANUTS I)^(16)^. This included detailed information on the participant’s dietary intake such as nutrient intake per meal/day, dairy intake, diet diversity, food habits as well as breast-feeding and complementary feeding information. Amino acid levels will be measured in a sub-sample of participants to further explore metabolomic profiles linked to dietary intake. Apart from measuring physical fitness, accelerometry, age-specific physical activity and sleep questionnaires were employed to assess objective data on physical activity, sedentary and sleep behaviour. These novel assessments are rarely conducted as part of national surveys and will further stimulate and broaden the development of targeted nutrition and lifestyle programs by governments and policy makers.

The large scale of SEANUTS II and the objective to compare the collected data in a pooled analysis, demand for high-end management of the study and strict alignment of protocols and operating procedures among the field staff. The level of complexity was greatly increased when the COVID-19 pandemic hit the region, in the midst of data collection.

During the first wave (March 2020) of COVID-19 infections in Southeast Asia, all countries put field work on hold between two to four months. Indonesia, Malaysia and Thailand had to stop recruitment and data collection prematurely, while Vietnam was not able to commence any activities. Following close monitoring of the pandemic situation, a decision was made for Indonesia and Malaysia to stop recruitment completely as the situation was logistically unsafe for data collection, although only ∼50 % and ∼80 % of recruitment was completed, respectively. In view of this, the data collected for Indonesia only represented twenty-one out of a total of forty-six regencies, of which fifteen regencies represented Java and Sumatra, while data for Malaysia represented only Peninsular Malaysia and did not include Sabah and Sarawak. Since the COVID-19 situation in Thailand was under control and deemed to be manageable, the team was able to re-start recruitment when the authorities deemed it safe for both the field team and the children and their families. However, data collection was only completed in thirty out of the forty-four districts. The team in Vietnam was able to start data collection in September 2020 and managed to finalise the original target number of participants despite major challenges (local floods and COVID-19 outbreaks).

Without any doubt, the COVID-19 pandemic entailed a significant challenge for the successful execution of SEANUTS II. In the midst of the crisis, the study teams managed to implement a COVID-19 questionnaire concerning the impact of the COVID-19 pandemic on nutrition and lifestyle factors. Moreover, a selection of questionnaires was repeated among previously recruited participants through an online survey platform or via telephone interviews in Indonesia and Malaysia, to compare some of the data pre- and during the respective periods of lockdown, especially for data on food intake/habits and physical activity.

As a result, SEANUTS II will not only deliver broad data on nutrition and lifestyle parameters of the childhood population in four Southeast Asian countries but will also significantly contribute to our understanding of the impact of the pandemic in the region. If we want to successfully reduce the prevalence of malnutrition in the coming years, a holistic view on the situation to date is of key importance. We are only beginning to understand the high burden on food supply, family income and reduced access to health care caused by the pandemic, resulting in reduced access to nutritious food and poor child and maternal health circumstances^(50)^. The initiatives to reduce malnutrition in the childhood population will need to overcome these detrimental changes caused by the global crisis.

We acknowledge that there are a few limitations in the study. Although this is a large-scale study with more than 13 000 subjects recruited, parents were being interviewed by researchers to fill in numerous questionnaires and this could lead to fatigue and thus inaccurate reporting. Second, the nutrient intake for children <2 years old was not well validated as we used estimations for breastmilk from literature. Lastly, data collection was conducted before as well as during the COVID-19 pandemic, for Indonesia, Malaysia and Thailand, while Vietnam data collection was done only during the pandemic. Therefore, comparisons among countries might not be fair, particularly for children’s physical activity, sun exposure and dietary habits.

### Conclusion

SEANUTS II will provide useful and relevant data that will inform future nutrition intervention strategies in the context of a global crisis that further compromises child well-being. Insights into nutrient deficiencies and inadequacies gained from blood biochemistry and dietary intake data will offer food industries in the region with relevant information to develop tailor-made food products that address current nutrition and health inadequacies. Data on impact of COVID-19 can be interpreted to provide timely interventions. Ultimately, we surmise that SEANUTS II will benefit the health and well-being of the childhood population in Southeast Asia.

## SEANUTS II study group

The SEANUTS II Study Group comprises the following: Universiti Kebangsaan Malaysia: Bee Koon Poh, Jyh Eiin Wong, Nik Shanita Safii, Nor MF Farah, Nor Aini Jamil, Razinah Sharif, Caryn Mei Hsien Chan, Swee Fong Tang, Lei Hum Wee, Siti Balkis Budin, Denise Koh, Abd.Talib Ruzita, Nur Zakiah Mohd Saat, Mohd Jamil Sameeha, A. Karim Norimah, See Meng Lim, Jasmine Siew Min Chia, Shoo Thien Lee. Universitas Indonesia: Rini Sekartini, Dian Novita Chandra, Aria Kekalih, Listya Tresnanti, Dina Indah, Ari Prayogo, Roselyne Anggraini, Luthfi Saiful Arif, Wahyu Budi Santoso, Saptawati Bardosono, Aryono Hendarto, Soedjatmiko. Mahidol University, Thailand: Nipa Rojroongwasinkul, Tippawan Pongcharoen, Siriporn Tuntipopipat, Nawarat Vongvimetee, Triwoot Phanyotha, Pornpan Sukboon, Chawanphat Muangnoi, Kemika Praengam, Pattanee Winichagoon, Emorn Udomkesmalee, Wiyada Thasanasuwan, Atitada Boonpraderm, Weerachat Srichan, Thitisan Tepthong Chayanist Wanijjakul, Thanisorn Rattanayoung, Kankunlanat Nimmannun, Yada Saokosol. National Institute of Nutrition, Vietnam: Nga Thuy Tran, Van Khanh Tran, Duong Thanh Tran, Tu Song Nguyen, Phuong Thi Lan Nguyen, Tu Tran Ngoc Nguyen, Anh The Nguyen, Hanh Thi Dang, An Vinh Pham, Anh Thi Van Nguyen, Bac Huu Nguyen, Tung Van Thanh Le, Ngan Thi Pham, Thoan Dieu Nguyen, Trang Thi Huyen Nguyen, Son Duy Nguyen, Ha Thu Nguyen, Phuong Thi Mai Tuan, Hoa Anh Le, Trung Duc Le. FrieslandCampina: Ilse Khouw, Swee Ai Ng, Marlotte M. Vonk, Serene Y. Tan, Ye Sun, Lucas Actis-Goretta, Panam Parikh, Friska Navisa Ratri (FrieslandCampina Indonesia), Miah Chua (DLMI Malaysia), Chumapa Deesudchit (FrieslandCampina Thailand), Huong Bui Thi Thu (FrieslandCampina Vietnam).
